# Bis(3-acetyl­pyridine-κ*N*)bis­(methanol-κ*O*)bis­(thio­cyanato-κ*N*)nickel(II)

**DOI:** 10.1107/S1600536812008860

**Published:** 2012-03-17

**Authors:** Julia Werner, Jan Boeckmann, Inke Jess, Christian Näther

**Affiliations:** a Institut für Anorganische Chemie, Christian-Albrechts-Universität Kiel, Max-Eyth-Strasse 2, 24118 Kiel, Germany

## Abstract

In the crystal structure of the title compound, [Ni(NCS)_2_(C_7_H_7_NO)_2_(CH_3_OH)_2_], the Ni^2+^ cations are coordinated by two thio­cyanate anions, two 3-acetyl­pyridine ligands and two methanol mol­ecules within slightly distorted NiN_4_O_2_ octa­hedra. The asymmetric unit consists of one Ni^2+^ cation, which is located on a center of inversion, as well as one thio­cyanate anion, one 3-acetyl­pyridine ligand and one methanol mol­ecule in general positions. The discrete complexes are linked by two pairs of O—H⋯O hydrogen bonds between the hy­droxy H atom and the acetyl O atom into chains along the *b* axis.

## Related literature

For general background information including details on thermal decomposition reactions and magnetic properties of the precursor and μ-1,3 bridging compounds, see: Näther & Greve (2003 ▶); Boeckmann & Näther (2010 ▶, 2011 ▶); Wöhlert *et al.* (2011 ▶). For a description of the Cambridge Structural Database, see: Allen (2002 ▶). 
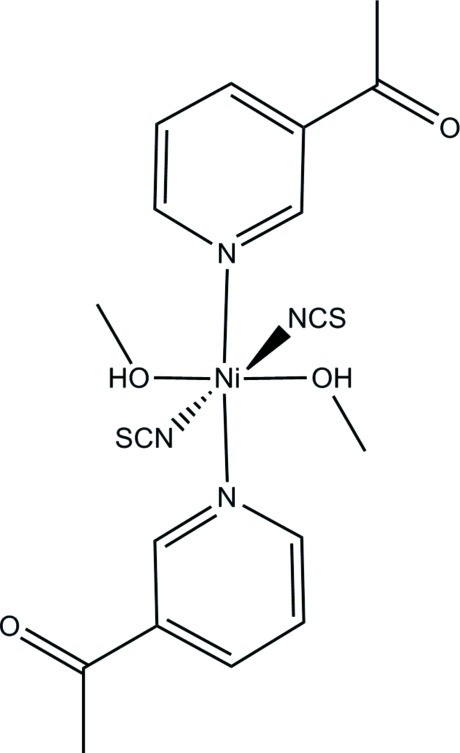


## Experimental

### 

#### Crystal data

[Ni(NCS)_2_(C_7_H_7_NO)(CH_4_O)_2_]
*M* *_r_* = 481.23
Monoclinic, 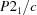

*a* = 7.7088 (7) Å
*b* = 14.6893 (9) Å
*c* = 9.6887 (8) Å
β = 96.782 (10)°
*V* = 1089.44 (15) Å^3^
*Z* = 2
Mo *K*α radiation
μ = 1.11 mm^−1^
*T* = 180 K
0.19 × 0.14 × 0.11 mm

#### Data collection

Stoe IPDS-1 diffractometer
Absorption correction: numerical (*X-SHAPE* and *X-RED32*; Stoe & Cie, 2008 ▶) *T*_min_ = 0.826, *T*_max_ = 0.881
9642 measured reflections
2555 independent reflections
2041 reflections with *I* > 2σ(*I*)
*R*_int_ = 0.060

#### Refinement

*R*[*F* ^2^ > 2σ(*F* ^2^)] = 0.039
*wR*(*F* ^2^) = 0.101
*S* = 0.99
2555 reflections
134 parameters
H-atom parameters constrained
Δρ_max_ = 0.41 e Å^−3^
Δρ_min_ = −0.66 e Å^−3^

### 

Data collection: *X-AREA* (Stoe & Cie, 2008 ▶); cell refinement: *X-AREA*; data reduction: *X-RED32* (Stoe & Cie, 2008 ▶); program(s) used to solve structure: *SHELXS97* (Sheldrick, 2008 ▶); program(s) used to refine structure: *SHELXL97* (Sheldrick, 2008 ▶); molecular graphics: *XP* in *SHELXTL* (Sheldrick, 2008 ▶); software used to prepare material for publication: *publCIF* (Westrip, 2010 ▶).

## Supplementary Material

Crystal structure: contains datablock(s) I, global. DOI: 10.1107/S1600536812008860/jj2122sup1.cif

Structure factors: contains datablock(s) I. DOI: 10.1107/S1600536812008860/jj2122Isup2.hkl

Additional supplementary materials:  crystallographic information; 3D view; checkCIF report

## Figures and Tables

**Table 1:** Selected geometric parameters (Å, °)

Ni1—N1	2.0357 (18)
Ni1—O21	2.0943 (14)
Ni1—N11	2.1154 (19)

**Table 2:** Hydrogen-bond geometry (Å, °)

*D*—H⋯*A*	*D*—H	H⋯*A*	*D*⋯*A*	*D*—H⋯*A*
O21—H1*O*⋯O11^ii^	0.84	1.87	2.700 (2)	172

Symmetry code: (ii) 
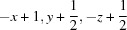
.

**Fig. 1.**
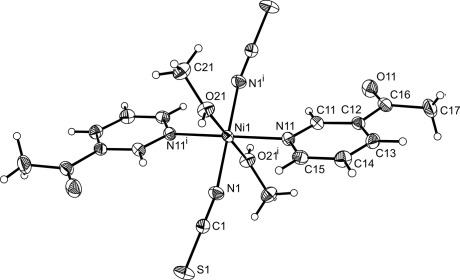
: Crystal structure of the title compound with labelling and displacement ellipsoids drawn at the 50% probability level. Symmetry code: i = -x+1,-y+1,-z+1.

**Fig. 2.**
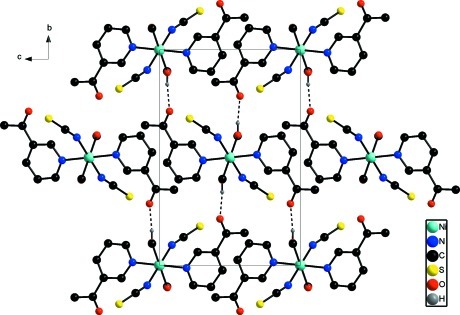
: Packing diagram of the title compound with view along the crystallographic a axis. Hydrogen bonding is shown as dashed lines and for clarity only the O-H H atoms are shown.

## supplementary crystallographic information

### Comment

The structure of the title compound was prepared within a project on the synthesis of transition metal coordination polymers containing µ-1,3 bridging thiocyanato anions and neutral N-donor co-ligands by thermal decomposition of suitable precursor compounds with N-terminal bonded anions (Boeckmann & Näther, 2010, 2011; Wöhlert *et al.*, 2011). In the preparation of a precursor compound using 3-acetylpyridine as co-ligand crystals of the title compound were obtained and characterized by single crystal x-Ray diffraction.

In the crystal structure the Nickel(II) cations are coordinated by four nitrogen atoms of two terminal N-bonded thiocyanato anions and two terminal bonded 3-acetylpyridine coligands as well as two methanol molecules, all of the related by symmetry into discrete complexes (Fig. 1). The coordination polyhedron of the Ni cations can be described as a slightly distorted octahedra with the Ni cation located on a centre of inversion (Table 1).

The discrete complexes are linked by two pairs of O—H···O hydrogen bonds between the hydroxy H atom and the acetyl O atom into chains, which are elongated in the direction of the crystallographic *b* axis (Fig. 2 and Table 2). It must be noted that according to a search in the CCDC database (ConQuest Ver.1.14.2012) (Allen, 2002) coordination compounds based on metal thiocyanates and 3-acetylpyridine are unknown.

### Experimental

Nickel thiocyanate and 3-acetylpyridine were purchased from Alfa Aesar. The title compound was prepared by the reaction of 174.9 mg Ni(NCS)_2_ (1.00 mmol) and 27.3 µ*L* 3-acetylpyridine (0.25 mmol) in 2 mL methanol at RT in a closed 3 ml snap cap vial. After three days colourless blocks of the title compound were obtained.

### Refinement

The C-H H atoms were positioned with idealized geometry and were refined isotropically with *U*_eq_(H) = 1.2 *U*_eq_(C) for aromatic H atoms (1.5 for methyl H atoms) using a riding model with C—H = 0.95 Å (aromatic) and with C—H = 0.98 Å (methyl). The O-H H atom was located in a difference map, its bond lengths set to ideal values of 0.84 Å and afterwards they were refined using a riding model with *U*~eq~(H) = 1.5 *U*~eq~(O)

### Crystal data

**Table:**

[Ni(NCS)_2_(C_7_H_7_NO)(CH_4_O)_2_]	*F*(000) = 500
*M**_r_* = 481.23	*D*_x_ = 1.467 Mg m^−^^3^
Monoclinic, *P*2_1_/*c*	Mo *K*α radiation, λ = 0.71073 Å
Hall symbol: -P 2ybc	Cell parameters from 9642 reflections
*a* = 7.7088 (7) Å	θ = 2.5–28.0°
*b* = 14.6893 (9) Å	µ = 1.11 mm^−^^1^
*c* = 9.6887 (8) Å	*T* = 180 K
β = 96.782 (10)°	Block, blue
*V* = 1089.44 (15) Å^3^	0.19 × 0.14 × 0.11 mm
*Z* = 2	

### Data collection

**Table:**

Stoe IPDS-1 diffractometer	2555 independent reflections
Radiation source: fine-focus sealed tube	2041 reflections with *I* > 2σ(*I*)
Graphite monochromator	*R*_int_ = 0.060
φ scans	θ_max_ = 28.0°, θ_min_ = 2.5°
Absorption correction: numerical (*X-SHAPE* and *X-RED32*; Stoe & Cie, 2008)	*h* = −10→10
*T*_min_ = 0.826, *T*_max_ = 0.881	*k* = −19→18
9642 measured reflections	*l* = −12→12

### Refinement

**Table:**

Refinement on *F*^2^	Secondary atom site location: difference Fourier map
Least-squares matrix: full	Hydrogen site location: inferred from neighbouring sites
*R*[*F*^2^ > 2σ(*F*^2^)] = 0.039	H-atom parameters constrained
*wR*(*F*^2^) = 0.101	*w* = 1/[σ^2^(*F*_o_^2^) + (0.0688*P*)^2^] where *P* = (*F*_o_^2^ + 2*F*_c_^2^)/3
*S* = 0.99	(Δ/σ)_max_ < 0.001
2555 reflections	Δρ_max_ = 0.41 e Å^−^^3^
134 parameters	Δρ_min_ = −0.66 e Å^−^^3^
0 restraints	Extinction correction: *SHELXL97* (Sheldrick, 2008), Fc^*^=kFc[1+0.001xFc^2^λ^3^/sin(2θ)]^-1/4^
Primary atom site location: structure-invariant direct methods	Extinction coefficient: 0.028 (3)

### Special details

**Table:**

Geometry. All esds (except the esd in the dihedral angle between two l.s. planes) are estimated using the full covariance matrix. The cell esds are taken into account individually in the estimation of esds in distances, angles and torsion angles; correlations between esds in cell parameters are only used when they are defined by crystal symmetry. An approximate (isotropic) treatment of cell esds is used for estimating esds involving l.s. planes.
Refinement. Refinement of F^2^ against ALL reflections. The weighted R-factor wR and goodness of fit S are based on F^2^, conventional R-factors R are based on F, with F set to zero for negative F^2^. The threshold expression of F^2^ > 2sigma(F^2^) is used only for calculating R-factors(gt) etc. and is not relevant to the choice of reflections for refinement. R-factors based on F^2^ are statistically about twice as large as those based on F, and R- factors based on ALL data will be even larger.

### Fractional atomic coordinates and isotropic or equivalent isotropic displacement parameters (Å^2^)

**Table:**

	*x*	*y*	*z*	*U*_iso_*/*U*_eq_	
Ni1	0.5000	0.5000	0.5000	0.01655 (14)	
N1	0.6742 (2)	0.59365 (12)	0.5873 (2)	0.0254 (4)	
C1	0.7710 (3)	0.62982 (13)	0.6700 (2)	0.0205 (4)	
S1	0.90677 (8)	0.68081 (4)	0.78668 (7)	0.03342 (18)	
N11	0.6038 (2)	0.51317 (11)	0.30841 (19)	0.0195 (4)	
O11	0.6251 (2)	0.28548 (11)	0.0710 (2)	0.0333 (4)	
C11	0.6169 (3)	0.43931 (14)	0.2295 (2)	0.0203 (4)	
H11	0.5778	0.3827	0.2619	0.024*	
C12	0.6841 (3)	0.44086 (14)	0.1032 (2)	0.0199 (4)	
C13	0.7383 (3)	0.52410 (16)	0.0541 (2)	0.0248 (5)	
H13	0.7830	0.5280	−0.0330	0.030*	
C14	0.7256 (3)	0.60105 (15)	0.1351 (3)	0.0265 (5)	
H14	0.7621	0.6586	0.1044	0.032*	
C15	0.6593 (3)	0.59317 (14)	0.2609 (2)	0.0228 (4)	
H15	0.6525	0.6462	0.3162	0.027*	
C16	0.6908 (3)	0.35298 (15)	0.0263 (2)	0.0249 (5)	
C17	0.7795 (4)	0.3502 (2)	−0.1025 (3)	0.0365 (6)	
H17A	0.7727	0.2884	−0.1408	0.055*	
H17B	0.9023	0.3677	−0.0800	0.055*	
H17C	0.7217	0.3927	−0.1711	0.055*	
O21	0.3202 (2)	0.60443 (10)	0.44475 (18)	0.0242 (3)	
H1O	0.3466	0.6599	0.4452	0.036*	
C21	0.1344 (3)	0.59987 (17)	0.4463 (3)	0.0311 (5)	
H21A	0.0818	0.6583	0.4156	0.047*	
H21B	0.1085	0.5868	0.5409	0.047*	
H21C	0.0862	0.5515	0.3835	0.047*	

### Atomic displacement parameters (Å^2^)

**Table:**

	*U*^11^	*U*^22^	*U*^33^	*U*^12^	*U*^13^	*U*^23^
Ni1	0.0195 (2)	0.01456 (19)	0.0153 (2)	−0.00132 (13)	0.00091 (13)	−0.00197 (13)
N1	0.0268 (9)	0.0240 (9)	0.0251 (11)	−0.0070 (7)	0.0017 (8)	−0.0042 (7)
C1	0.0229 (10)	0.0166 (9)	0.0223 (11)	−0.0008 (7)	0.0047 (8)	0.0015 (7)
S1	0.0345 (3)	0.0316 (3)	0.0306 (3)	−0.0083 (2)	−0.0113 (2)	−0.0015 (2)
N11	0.0215 (9)	0.0202 (8)	0.0167 (9)	0.0005 (6)	0.0018 (7)	0.0007 (6)
O11	0.0400 (10)	0.0245 (8)	0.0364 (10)	0.0004 (7)	0.0085 (8)	−0.0078 (7)
C11	0.0229 (10)	0.0192 (9)	0.0183 (11)	0.0000 (7)	0.0004 (8)	−0.0006 (8)
C12	0.0190 (9)	0.0224 (10)	0.0176 (10)	0.0017 (7)	−0.0003 (7)	−0.0003 (8)
C13	0.0238 (11)	0.0308 (11)	0.0202 (11)	−0.0013 (8)	0.0040 (8)	0.0046 (9)
C14	0.0285 (11)	0.0226 (10)	0.0285 (12)	−0.0024 (8)	0.0035 (9)	0.0050 (9)
C15	0.0239 (10)	0.0192 (10)	0.0249 (12)	−0.0024 (8)	0.0010 (8)	−0.0003 (8)
C16	0.0244 (10)	0.0274 (11)	0.0218 (11)	0.0039 (8)	−0.0013 (8)	−0.0046 (8)
C17	0.0398 (13)	0.0476 (15)	0.0224 (13)	0.0060 (11)	0.0044 (10)	−0.0088 (11)
O21	0.0221 (7)	0.0184 (7)	0.0318 (9)	0.0034 (5)	0.0019 (6)	0.0038 (6)
C21	0.0231 (11)	0.0335 (12)	0.0368 (14)	0.0041 (9)	0.0046 (9)	0.0042 (10)

### Geometric parameters (Å, º)

**Table:**

Ni1—N1^i^	2.0357 (18)	C13—C14	1.386 (3)
Ni1—N1	2.0357 (18)	C13—H13	0.9500
Ni1—O21^i^	2.0943 (14)	C14—C15	1.380 (3)
Ni1—O21	2.0943 (14)	C14—H14	0.9500
Ni1—N11	2.1154 (19)	C15—H15	0.9500
Ni1—N11^i^	2.1154 (19)	C16—C17	1.493 (4)
N1—C1	1.157 (3)	C17—H17A	0.9800
C1—S1	1.629 (2)	C17—H17B	0.9800
N11—C11	1.338 (3)	C17—H17C	0.9800
N11—C15	1.350 (3)	O21—C21	1.435 (3)
O11—C16	1.216 (3)	O21—H1O	0.8399
C11—C12	1.384 (3)	C21—H21A	0.9800
C11—H11	0.9500	C21—H21B	0.9800
C12—C13	1.394 (3)	C21—H21C	0.9800
C12—C16	1.494 (3)		
			
N1^i^—Ni1—N1	180.00 (13)	C14—C13—H13	120.7
N1^i^—Ni1—O21^i^	89.75 (7)	C12—C13—H13	120.7
N1—Ni1—O21^i^	90.25 (7)	C15—C14—C13	119.3 (2)
N1^i^—Ni1—O21	90.25 (7)	C15—C14—H14	120.3
N1—Ni1—O21	89.75 (7)	C13—C14—H14	120.3
O21^i^—Ni1—O21	180.00 (9)	N11—C15—C14	122.6 (2)
N1^i^—Ni1—N11	89.80 (7)	N11—C15—H15	118.7
N1—Ni1—N11	90.20 (7)	C14—C15—H15	118.7
O21^i^—Ni1—N11	89.06 (7)	O11—C16—C17	121.8 (2)
O21—Ni1—N11	90.94 (7)	O11—C16—C12	119.1 (2)
N1^i^—Ni1—N11^i^	90.20 (7)	C17—C16—C12	119.1 (2)
N1—Ni1—N11^i^	89.80 (7)	C16—C17—H17A	109.5
O21^i^—Ni1—N11^i^	90.94 (7)	C16—C17—H17B	109.5
O21—Ni1—N11^i^	89.06 (7)	H17A—C17—H17B	109.5
N11—Ni1—N11^i^	180.0	C16—C17—H17C	109.5
C1—N1—Ni1	159.66 (19)	H17A—C17—H17C	109.5
N1—C1—S1	179.8 (2)	H17B—C17—H17C	109.5
C11—N11—C15	117.6 (2)	C21—O21—Ni1	126.46 (13)
C11—N11—Ni1	119.28 (14)	C21—O21—H1O	106.7
C15—N11—Ni1	123.16 (15)	Ni1—O21—H1O	123.8
N11—C11—C12	123.58 (19)	O21—C21—H21A	109.5
N11—C11—H11	118.2	O21—C21—H21B	109.5
C12—C11—H11	118.2	H21A—C21—H21B	109.5
C11—C12—C13	118.3 (2)	O21—C21—H21C	109.5
C11—C12—C16	117.70 (19)	H21A—C21—H21C	109.5
C13—C12—C16	124.0 (2)	H21B—C21—H21C	109.5
C14—C13—C12	118.6 (2)		

Symmetry code: (i) −*x*+1, −*y*+1, −*z*+1.

### Hydrogen-bond geometry (Å, º)

**Table:**

*D*—H···*A*	*D*—H	H···*A*	*D*···*A*	*D*—H···*A*
O21—H1*O*···O11^ii^	0.84	1.87	2.700 (2)	172

Symmetry code: (ii) −*x*+1, *y*+1/2, −*z*+1/2.
